# Chalcone derivatives' interaction with human serum albumin and cyclooxygenase-2†

**DOI:** 10.1039/d3ra07438b

**Published:** 2024-01-17

**Authors:** Subramani Karthikeyan, Ayyavu Thirunarayanan, Leon Bernet Shano, Arasappan Hemamalini, Anandh Sundaramoorthy, Rajendiran Mangaiyarkarasi, Norhidayah Abu, Singaravelu Ganesan, Shanmugavel Chinnathambi, Ganesh N. Pandian

**Affiliations:** a Centre for Healthcare Advancement, Innovation and Research, Vellore Institute of Technology Chennai 600 127 India s.karthikeyan@vit.ac.in; b Institute for Integrated Cell-Material Sciences, Institute for Advanced Study, Kyoto University Kyoto 616-8510 Japan chinnathambi.shanmugavel.8s@kyoto-u.ac.jp namasivayam.ganeshpandian.5z@kyoto-u.ac.jp; c Department of Chemical Engineering Biotechnology and Materials, FCFM, University of Chile Av. Beauchef 851 Santiago Chile; d Division of Physics, School of Advanced Sciences, Vellore Insititute of Technology (VIT) Chennai Campus Vandalur-Kelambakkam Road Tamil Nadu 600127 India; e Department of Chemistry, Government College of Engineering Srirangam Sethurapatti Thiruchirappalli Tamil Nadu India; f Department of Medical Physics, Anna University Chennai-600 025 India; g Centre for Nanotechnology Research, Vellore Institute of Technology Vellore Tamil Nadu India; h Department of Medical Microbiology & Parasitology, School of Medical Sciences, Universiti Sains Malaysia Health Campus, Kubang Kerian 16150 Kelantan Malaysia

## Abstract

Chalcone derivatives are an extremely valuable class of compounds, primarily due to the keto-ethylenic group, CO–CH=CH–, they contain. Moreover, the presence of a reactive α,β-unsaturated carbonyl group confers upon them a broad range of pharmacological properties. Recent developments in heterocyclic chemistry have led to the synthesis of chalcone derivatives, which have been biologically investigated for their activity against certain diseases. In this study, we investigated the binding of new chalcone derivatives with COX-2 (cyclooxygenase-2) and HSA (Human Serum Albumin) using spectroscopic and molecular modeling studies. COX-2 is commonly found in cancer and plays a role in the production of prostaglandin E (2), which can help tumors grow by binding to receptors. HSA is the most abundant protein in blood plasma, and it transports various compounds, including hormones and fatty acids. The conformation of chalcone derivatives in the HSA complex system was established through fluorescence steady and excited state spectroscopy techniques and FTIR analyses. To gain a more comprehensive understanding, molecular docking, and dynamics were conducted on the target protein (COX-2) and transport protein (HSA). In addition, we conducted density-functional theory (DFT) and single-point DFT to understand intermolecular interaction in protein active sites.

## Abstract 2

In this study, various spectroscopic and computational techniques were utilized to investigate how new chalcone derivatives bind with cyclooxygenase-2 (COX-2) and Human Serum Albumin (HSA).

## Graphical abstract

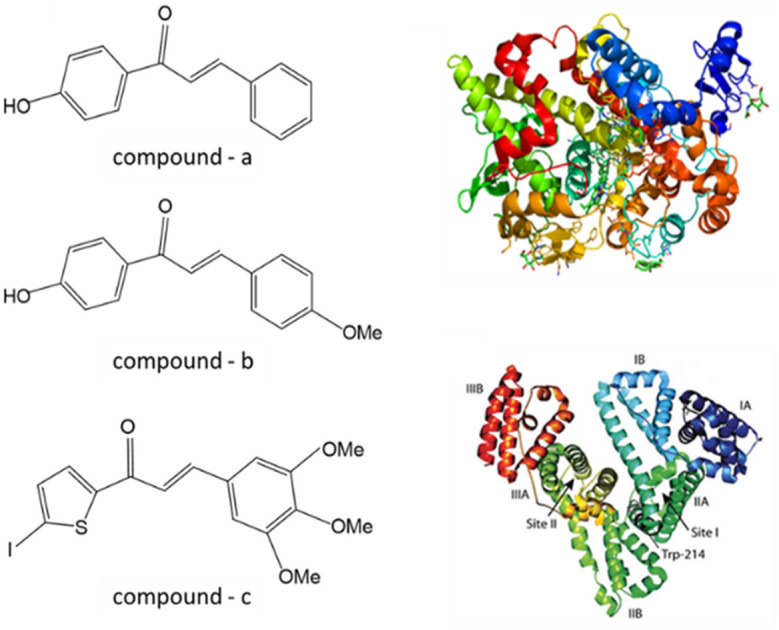

## 1. Introduction

Chalcone, a naturally occurring chemical compound in plants like teas, vegetables, and fruits, offers numerous health benefits.^1^ Chalcone derivatives possess replaceable hydrogens, making them a favored raw material for synthetic chemists.^2–6^ Due to their uncomplicated chemical structures, ease of synthesis, and multiple modification sites in the skeletons, flavonoids have been employed as potent agents for various ailments, including anti-inflammatory, anti-diabetic, antiplatelet and antimicrobial, anticancer antioxidants, and anti-allergic treatments.^7–9^ Usually, natural products and their derivatives are crucial in treating various illnesses.^10–12^ For instance, chalcone-loaded carbon nanomaterials are utilized as anticancer and bioimaging agents.^13^ In addition, the chalcone family has demonstrated potential against cancer through multiple mechanisms, such as cell cycle disruption, apoptosis induction, immunomodulation, autophagy regulation, and inflammation modulation. The chalcone derivatives exhibit anti-inflammatory properties by regulating the expression of genes associated with COX-2 (cyclooxygenase-2), inducible nitric oxide synthase, and various crucial cytokines.^14–17^ Recent research has highlighted the significance of chalcones as anti-inflammatory agents that effectively prevent cell migration and the production of TNF.^18^ In addition, chalcones are highly promising anti-inflammatory drugs for treating immunological and inflammatory conditions, and their unique chemical structures make them an ideal candidate for enhancing drug formulation and development.^19–23^

**Fig. 1:**
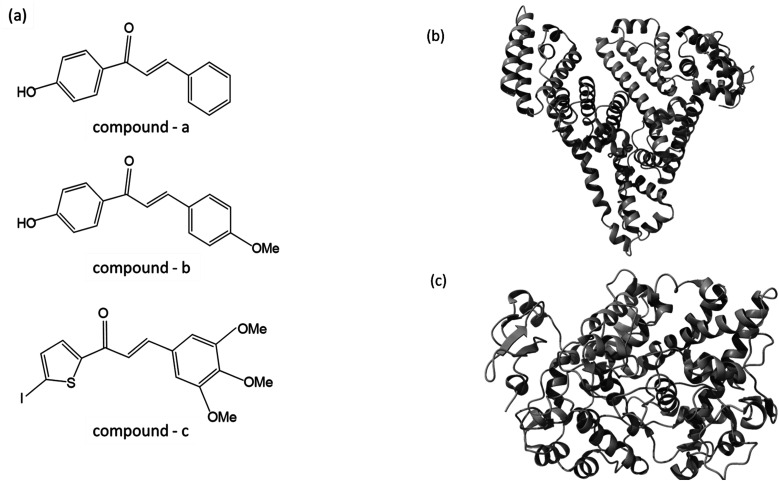
(a) The chemical structure of chalcone derivatives, (b) a crystal structure of a COX-2 chain complex with the drug celecoxib, and (c) the crystal structure of HSA.

As part of our ongoing effort to find effective anti-inflammatory treatments, we have synthesized a series of new chalcone compounds. Our research has allowed us to predict the effectiveness of these compounds in regulating the immune system, which we hope will lead to new treatments for a variety of inflammatory conditions. The primary goal of this study was to gain comprehensive insights into the binding properties of novel chalcone derivatives in both the transport protein (HSA) and the target protein (COX-2) by employing a diverse range of biophysical techniques (Fig. 1). The interaction between proteins and drugs has received significant attention from the research community. One primary protein in blood plasma is serum albumin, which plays a crucial role in regulating blood pH and colloidal osmotic pressure. Moreover, albumin is responsible for transporting various substances, such as fatty acids, amino acids, and drugs. There are two identified isoforms of COX, namely COX-1 and COX-2. These isoforms were named in the order of their discovery and are responsible for this crucial conversion process.^24^ COX-2 is an enzyme responsible for catalyzing the conversion of arachidonic acid into various types of lipid mediators, including prostaglandins. The COX-2 gene is located on chromosome 1q25.2-25.3 and consists of 10 exons and 9 introns. A chalcone derivative has demonstrated its potential as an anticancer agent, with COX-2 being identified as one of its targets.^25^ Several types of cancers, like breast, gastric, colon, lung, esophageal, and prostate, exhibit an elevated expression of the COX-2 isozyme. Several parameters, including age, tumor size, high grade, negative hormone receptor status, and high proliferation rate, are correlated with up to 40% expression of COX-2 enzyme in human breast cancer. In colorectal cancer, COX-2 expression is found to be upregulated by interleukin-1b *via* multiple pathways.^26,27^

In this work, we have selected these two proteins to interact with newly synthesized chalcone derivatives. The fluorescence spectroscopic method is a rapid and sensitive technique used to probe changes in the protein's environment. It helps to understand the binding mechanism and nature of binding. In the present study, we employed various fluorescence spectroscopic techniques (emission, synchronous, excitation-emission matrix, TRES, fluorescence resonant energy transfer) in addition to UV-visible, fourier transform infrared spectroscopy (FT-IR). In addition, molecular modeling, molecular dynamics, and DFT calculations unravel the intricacies of how proteins interact with chalcone derivatives.

## 2. Materials and methods

### 2.1 Method for synthesizing (*E*)-1-(4-hydroxyphenyl)-3-phenylprop-2-en-1-one: (compound 1)

Hydroxyl chalcone 1 was synthesized using the Claisen–Schmidt condensation reaction. Stir the mixture of benzaldehyde (1.0 equiv.) and 4-hydroxy acetophenone (1.0 equiv.) in 100 mL of anhydrous ethanol and 10% NaOH solution (50 mL) for 12 hours at ambient temperature. To achieve the desired result, it is recommended to utilize diluted HCl to neutralize the reaction mixture. After neutralization, carefully pour the mixture through ice water, measuring 100 mL. After the precipitation, the substance was washed and filtered with water and then meticulously dried until it was utterly moisture-free. The compound was recrystallized with ethanol, resulting in the formation of light-yellow crystals. (yield 92%, m.p. 132 °C).^28–30^ The spectral data and copies of corresponding spectra for the synthesized compounds have been previously elucidated in our earlier studies.^31–36^

### 2.2 Method for synthesizing (*E*)-1-(4-hydroxyphenyl)-3-(4-methoxyphenyl) prop-2-en-1-one – 2: (compound 2)

Hydroxyl Chalcone 2 was synthesized through Claisen–Schmidt condensation reaction of *p*-anisaldehyde (1.0 equiv.), with 4-hydroxy acetophenone (1.0 equiv.) 12 hours of stirring at room temperature were performed in 100 mL of anhydrous ethanol and 50 mL of 10% NaOH solution. After the reaction, the mixture was poured into a container with 100 mL of ice water and then neutralized with dilute HCl. After forming the precipitate, it was washed and filtered rigorously using water and then dried thoroughly. It is imperative to utilize ethanol for the compound to undergo recrystallization, forming light-yellow crystals (yield 93%, m.p. 184–186 °C).^28,29,31–36^

### 2.3 Method for synthesizing (*E*)-1-(5-iodothiophen-2-yl)-3-(3,4,5-trimethoxyphenyl) prop-2-en-1-one – 4: (compound 3)

The Iodochalcone 3 was synthesized by Claisen–Schmidt condensation for 2-acetyl,5-iodo-thiophene (1.0 equiv.) with 3,4,5-trimethoxybenzaldehyde (1.0 equiv.) in 100 mL of anhydrous ethanol and 10% NaOH solution (50 mL), mixed at ambient temperature for 12 hours. To initiate the process, we added the solution to 100 milliliters of cold water and then treated it with a diluted hydrochloric acid. The crude product was obtained by filtering, repeatedly washing, and drying the precipitated product. The iodochalcone was purified through recrystallization in ethyl alcohol, resulting in a dark brown color and a clean appearance (yield 82%, m.p. 152 °C).^28,29,31–36^

### 2.4 Biological solution and reagents

The HSA molecule was obtained from Sigma Aldrich, and the HSA protein sample was used for experiments without undertaking any further purification steps. This allowed me to use the HSA protein sample naturally without altering its chemical composition or purity level. To achieve micromolar concentration, the HSA was prepared by diluting it in PBS with a pH of 7.4 using distilled water. When the concentration was at 10 M, the HSA absorption reached its maximum at a wavelength of 278 nm. The drugs were diluted in ethanol to a concentration of 20 mM (5 mg mL^−1^), and then further diluted into various μM concentrations.

### 2.5 Binding studies confirmation with spectroscopy techniques

We obtained UV-vis absorption spectra of the HSA and HSA-bound chalcone derivative complex ranging from 200 to 500 nm, and the wavelength maxima were around 276 nm (PerkinElmer Lamda35, Waltham, MA). The steady-state emission spectroscopic analysis was conducted using Fluorolog-3 (ISA, Jobin-Yvon-Spex, Edison, NJ). The slit width for excitation and emission was consistently maintained at 5 nm. As a result, it was determined that the protein molecule's excited wavelength and emission maxima were in close proximity to 280 nm to 350 nm. We recorded complete spectra of free protein and protein–drug complexes within the wavelength range of 300 nm to 500 nm.

The level of HSA titration was kept consistent at five μM. However, the chalcone derivative concentration for the HSA–chalcone derivative complex varied from 0 to 25 μM, with a two μM interval between each concentration. Through the utilization of Stern–Volmer eqn (1) and (2), we were able to establish the binding mechanism and reasoning behind the reduction in fluorescence intensity when incorporating chalcone derivatives into the HSA molecular environment.^37^

1 *F*_0_/*F* = 1 + *k*_q_*τ*_0_ [drug] = 1 + *k*_SV_ [drug]

2 *F*_corr_ = *F*_obs_. e^(*A*_1_ + *A*_2_)/2^

In biomolecule processes, the quenching rate constant is represented by *k*_q_, while the Stern–Volmer binding constant is represented by *k*_sv_. *τ*_0_ denotes the average lifetime of the tyr and trp residues that are commonly found in protein molecules. Accordingly, the values *A*_1_ and *A*_2_ indicate the absorbance of each constituent at the emission and excitation wavelengths. The variables *F*_obs_ and *F*_corr_ denote the fluorescence levels before and after correction, respectively, at the emission wavelength. By applying eqn (3), it is possible to calculate the binding parameter and the number of binding sites for chalcone derivatives within protein molecular environments.^38^

3 log[(*F*_0_–*F*)/*F*] = log  *k*_b_ + *n* log[drug]

In this equation, “*F*_0_” and “*F*” stand for the HSA fluorescence intensities with and without chalcone derivatives. The number of molecules involved (*n*) in a protein–drug complex's binding stoichiometry is determined by the binding constant (*k*_b_). In addition to calculating the binding free energy, Δ*G*° to protein, drug complexes were calculated using eqn (4).

4 Δ*G*° = −*RT* ln *k*_b_

The binding constant for the HSA–chalcone derivative complex is represented by *k*_b_, while *R* represents the gas constant (1.987 cal mol^−1^ k^−1^), and *T* represents the temperature (292 K, 298 K, 303 K). Furthermore, the Fluorolog-3 instrument was used to record the excitation-emission matrix of the HSA–chalcone and the free HSA derivative complex. This was done in the wavelength range of 230 nm to 500 nm while maintaining a slit width of 5 nm for both excitation and emission. In addition, the synchronous spectrum was recorded for both the protein–drug complex and the free protein. The cutoff range for tyr and trp residues was set at 15 nm and 60 nm, respectively.

A time-correlated single photon counting system (TCSPC), Fluorolog-3, was used to measure protein–drug complex and free protein lifetime. We used a Nano LED Source with a wavelength of 280 nm and a pulse width of less than 1 ns to record the lifetime decay profile of free protein and protein drug complex. A PMT detector then captured the data. The fluorescence emission decay profiles for protein molecules were measured at a 90-degree angle from the direction of the exciting light source. After amplifying the signal with a pulsed amplifier (TB-02, Horiba), we supplied it to a single channel fraction timing discriminator using Type No. 6915 from Philips Scientific in Mahwah, NJ. We utilized a TAC (time-to-time amplitude converter) that detected the first photon until the 350 nm emission signal peak reached 1000 counts. Before experimenting on protein–drug interaction decay, they measured the instrument response using Ludox-40 reagents in the 280 nm excitation wavelength range. The final result was analyzed with Decay Analysis Software (DAS6 v6.0, Horiba), and chi-square values were utilized to verify the linearity of the decay profile fitted in the protein–drug combination. To determine the average lifetime value, we use eqn (5).5
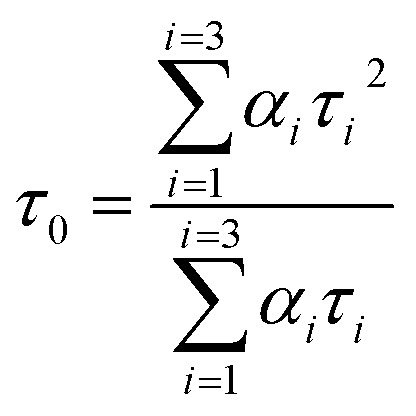


We conducted FTIR analysis on both free protein and protein–drug complex using JASCO model no IR/FT 6000 series. The experiment was conducted at an ambient temperature of 298 K, and we utilized ATR mode. Before the experiment, we removed background noise, reduced CO_2_, and resolved baselines for each sample. We recorded data within the wavelength range of 600 nm to 4000 nm and collected the output for all samples using a TGS detector.

Förster resonance energy transfer between HSA and chalcone derivative was analyzed using eqn (6) of the specific Förster theory.6
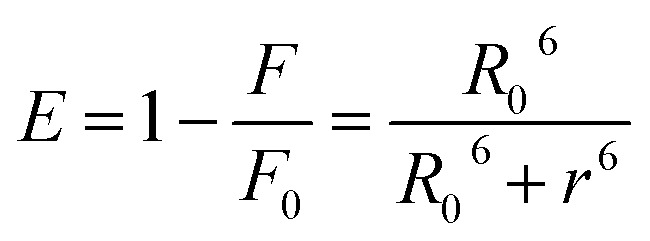


To determine the critical energy distance, *R*_0_, eqn (7) can be used, which considers the values for *E* − energy transfer efficiency, *r* – distance, and the presence or absence of chalcone derivative represented by *F* and *F*_0,_ respectively.

7 *R*_0_^6^ = 8.79 × 10^−25^[*k*^2^*N*^−4^*φ*_*D*_*J*]

The variable *k*^2^ represents the dipole spatial orientation factor, while *N* represents the refractive index (1.336). *φ*_D_ indicates the quantum yield (0.198), and *J* refers to the overlapping integral for the acceptor and donor of the HSA chalcone derivative complex.^39^8
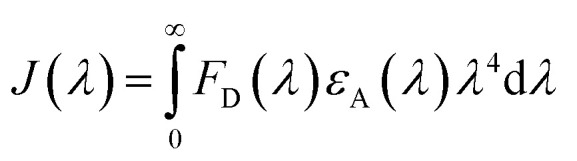


The fluorescence intensity of the HSA donor is represented by *F*_D_ (*λ*), while the chalcone derivative acceptor's molar extension coefficient is denoted by *ε*_A_ (*λ*).

### 2.6 Computational techniques

ChemSketch ACD software is used to draw the chemical structures of compounds A, B, and C. Then, the conjugate gradient algorithm was used to minimize those structures energetically, cycling through them 5000 times to achieve the most energetically favorable conformations. I have retrieved the protein structures for HSA (PDB ID: 4L8U) and COX-2 (PDB ID: 3LN1) from the protein data bank. The molecule COX-2 was chosen as a target through investigations of SAR (structural activity relationships) for these compounds using a Swiss target prediction server.^40^ The transport and target proteins experienced pre-processing using the Protein Prep Wizard panel to eliminate undesired crystal water molecules and heteroatoms. Notably, the protein 4L8U, obtained from the PDB, inherently contains amino acids in its structure. However, during the pretreatment process, we specifically removed 9-amino camptothecin, which is an amino acid, from the structure. The structure underwent optimization and minimization procedures using the OPLS2005 force field.^41^ For the docking studies, we extracted the native binding sites of both protein molecules. We conducted an induced fit docking (IFD) module within Schrödinger 2015, a flexible docking strategy between protein and compound-complex, resulting in 20 different best binding poses for both complexes.^42–44^ The optimal pose was selected for molecular dynamics simulation based on glide energy.

We conducted molecular dynamics studies on target and transport proteins in conjunction with compound c complex, running a 50 ns simulation. Isotopically charged ions were introduced into a TIP3P water solvation system buffer to achieve a balanced Ewald charge summation for the solvated protein entity. To reduce the system, we utilized a maximum of 5000 steps for iterations and employed a gradient convergence criterion of approximately 1.0 kcal mol^−1^. After being reduced, the system undergoes the Newtonian dynamics of the model system to calculate its energy. The simulation was recorded in 2 picosecond intervals using integration. Before initiating the molecular dynamics simulation, I conducted a six-stage relaxation process with the NPT ensemble default. Initially, the ensemble's solute-restrained Brownian dynamics were performed by maintaining an energy constant under the *NVT* condition. During the second stage, the *NVT* (canonical) ensemble was cooled down using a Berendsen thermostat, and a velocity equivalent to one pulse per second was applied to a non-hydrogen solute sample. After switching from an *NVT* ensemble to an NPT ensemble using a Berendsen barostat, the system maintained a pressure of 1 atm. To ensure system stability, a 1 ns equilibration was performed. The ensemble needed to be exposed to 50 ns to run molecular dynamics simulations.^45^

DFT (Density functional theory) calculations were conducted for the free compound c, and single point DFT was executed for the compound in the transport and target protein's active binding sites. To optimize and establish a single reference point, the B3LYP method (Becke3-Lee-Yang-Parr) was utilized. JAGUAR comprehensively evaluates all DFT surface parameters.^46^

## 3. Results and discussion

### 3.1 Experimental binding analysis

**Fig. 2:**
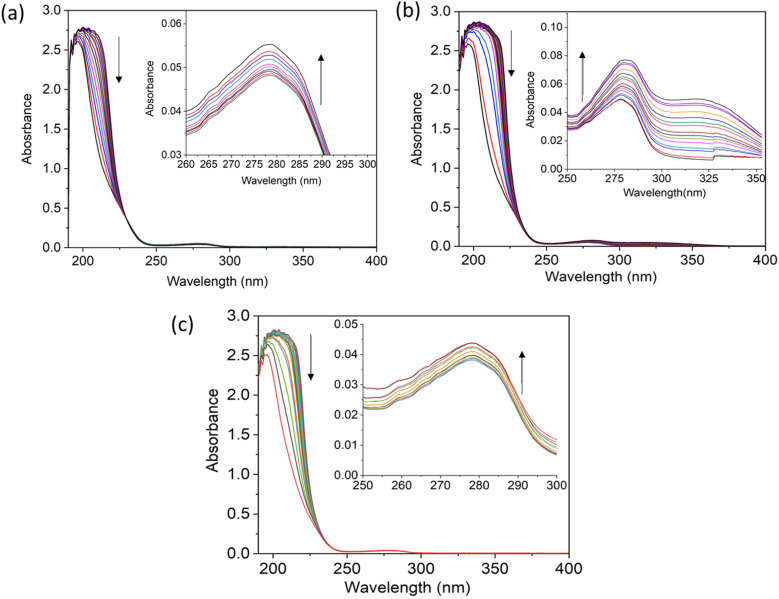
The UV-vis absorption spectra for compound A (a), compound B (b), and compound C (c) when complexed with HSA. The addition of drugs caused a decrease in absorbance in the polypeptide and aromatic amino acid regions, indicated respectively by upward and downward arrows.

The UV-vis method is commonly used to examine the interactions between proteins and drugs.^47^ The UV-vis absorption spectra of the HSA complex and free HSA (at a concentration of 1 μM) were examined in the presence of chalcone derivative compounds a, b, and c. The concentrations of compound a ranged from 0.5 to 5 μM at intervals of 0.5 μM, while those of compound b ranged from 0.5 to 7.5 μM at intervals of 0.5 μM. Compound c was tested at concentrations ranging from 0.5 to 5 μM, also at intervals of 0.5 μM. As seen in Fig. 2, the absorption spectrum of the HSA-chalcone derivative complex is displayed. After examining the figure, it was noted that there is no displacement in the peak wavelength of HSA within the chalcone derivatives complex system. Fig. 2b clearly demonstrates an undeniable increase in wavelength around 325 nm upon raising the concentration of compound b. On the other hand, it is evident that all compound-HSA complexes experienced a significant decrease in wavelength around 190–220 nm. The overall result suggests the presence of all three compounds in the HSA complex system, and there are possibilities for ground state formation between proteins in three compound complex systems.

**Fig. 3:**
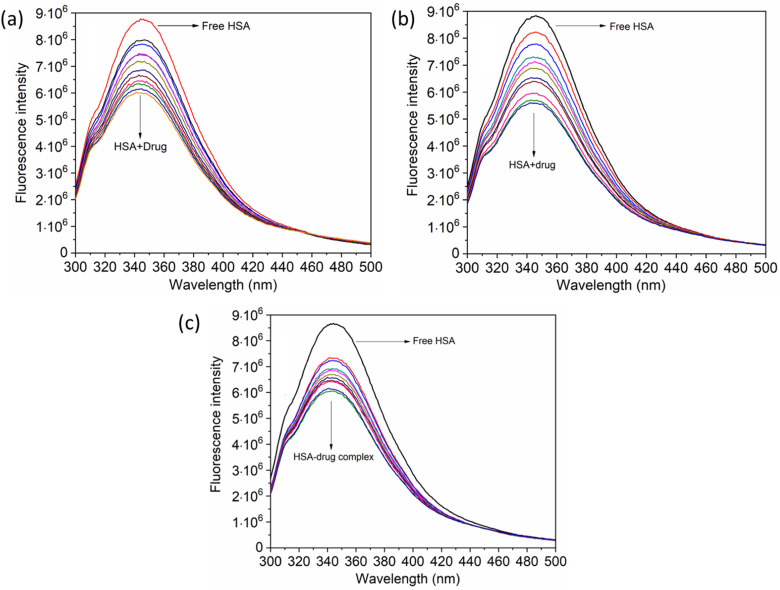
The steady-state emission spectra for compound A (a), compound B (b), and compound C (c) complexed with HSA. The horizontal arrow indicates free protein emission spectra, while the downward arrow indicates fluorescence quenching after the sequential addition of drugs.

When investigating protein–drug complexes, the fluorescence emission technique is typically the most effective method for exploring their binding mechanism.^48,49^ In Fig. 3, we can see the emission spectrum of the stable state of the HSA–chalcone derivative complex system. Notably, the maximum emission of HSA occurs at approximately 345 nm due to the tryptophan residue present in the system. When the concentration of the chalcone derivative compound is increased in the HSA complex system, the emission maximum of HSA gradually decreases without any shift for all three compounds. This suggests that the compound may be present in the HSA complex system.

**Fig. 4:**
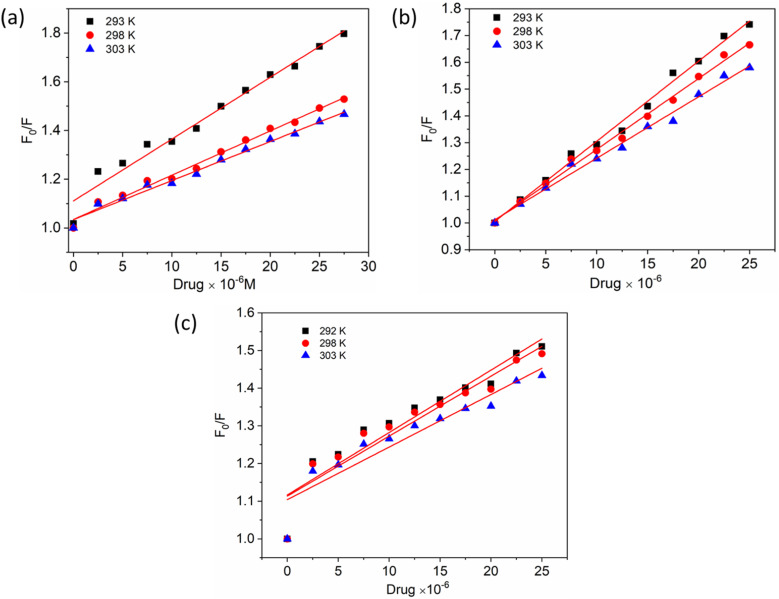
The Stern–Volmer plots for the complexes of compounds A (a), B (b), and C (c) with HSA were plotted at three different temperatures.

To gain a better understanding of the quenching mechanism in the HSA–chalcone derivative complex system, a Stern–Volmer plot was generated at three distinct temperatures: 292 K, 298 K, and 303 K (Fig. 4). According to scientific reports, quenching can be categorized as either dynamic or static, depending on whether the average quenching constant value falls above or below the limit of 2 × 10^10^ L mol^−1^ s^−1^. Values exceeding the limit are considered static, while those below it are classified as dynamic quenching.^50^ In Table 1, the determined quenching constant *k*_q_ values for the HSA–chalcone derivative complex system are higher than the average quenching constant limit. This indicates that the system belongs to static quenching. The data suggest that the complex system is efficiently quenched.

**Table 1:** Fluorescence binding parameters for HSA–chalcone derivatives (pH-7.4)

Compounds	*T* (K)	*k*_q_ (×10^18^ L mol^−1^ s^−1^)	*k*_b_ (×10^6^ L mol^−1^)	*n*	Δ*H*° (kJ mol^−1^)	Δ*G*° (kJ mol^−1^)	Δ*S*° (J mol^−1^ K^−1^)
Compound A	293	6.91	3.26	1.02	−188.05	−55.11	18.02
	298	3.96	2.90	1.05		−56.05	
	303	3.57	2.66	1.08		−56.99	
Compound B	293	5.20	4.43	0.93	−141.70	−44.16	15.38
	298	4.76	4.23	0.95		−41.81	
	303	4.26	4.17	1.01		−42.45	
Compound C	293	5.71	1.57	0.98	−184.10	−45.05	14.75
	298	5.53	1.40	1.41		−45.79	
	303	4.95	1.33	1.03		−46.53	

**Fig. 5:**
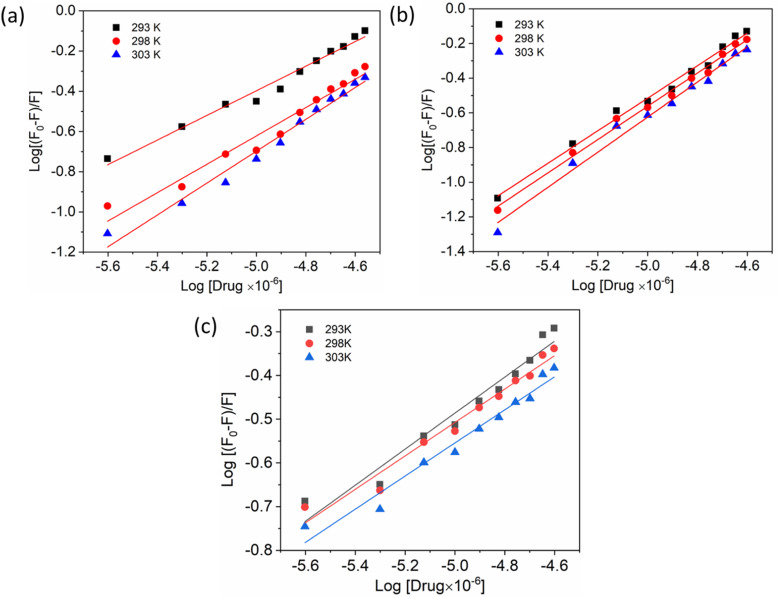
Double logarithmic plots show the interaction of compounds A (a), B (b), and C (c) with HSA at varying temperatures.

**Fig. 6:**
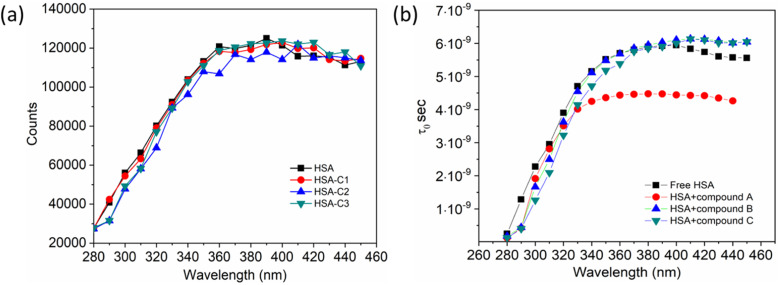
(a). The average photon counts for free HSA and HSA complexed with compounds A (C1), B (C2), and C (C3), as determined by TRES. (b). Calculated average lifetime value for HSA when it is either free or complexed with three compounds.

**Fig. 7:**
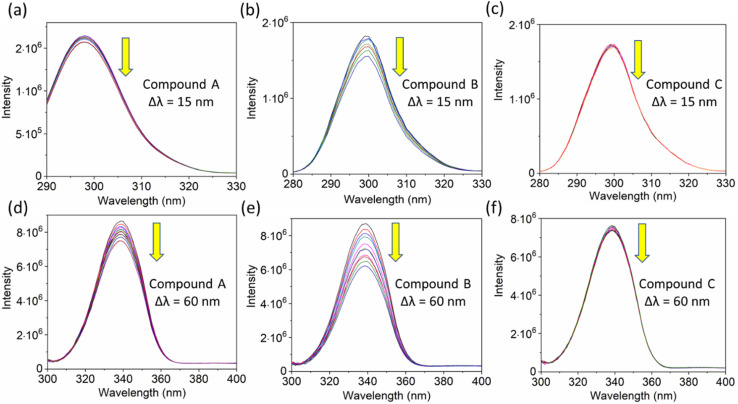
The synchronous fluorescence spectra of HSA with a Δ*λ* of 15 nm (Tyrosine, a–c) and Δ*λ* of 60 nm (Tryptophan, d–e) interaction with compounds A, B, and C. The downward arrow indicates synchronous fluorescence quenching after the sequential addition of drugs.

Additionally, a double logarithmic plot (Fig. 5) was computed to ascertain the binding constant, and the findings were recorded in Table 1. During the interaction of the chalcone derivative with the HSA complex system, the binding site remained at approximately one. Time-resolved emission spectroscopy was analyzed to confirm the quenching mechanism in the protein–drug complex system.^51–53^ Many articles commonly analyze TRES by only considering the lifetime decay curve for the protein–drug complex at the emission maxima of 350 nm. However, this article aims to investigate the stability of our newly synthesized chalcone derivative in the HSA complex system by exploring the excitation wavelength region between 280 nm and 440 nm using a 280 nm LED source. In the graph displayed in Fig. 6a, it is evident that the average photon counts of the free HSA and the HSA–chalone derivative complex in a 1 : 5 μM ratio differ significantly, with compound b showing a distinct variation from the average photon counts of free HSA. However, the other two compounds display only a slight difference. In calculating the average lifetime value depicted in Fig. 6b, it is evident that the compound displays a distinct quenching effect in comparison to the other two. It is possible that the varying functional groups in each compound contribute to the variations observed in the HSA complex system. The overall analysis suggests that the quenching confirmation of each compound in the HSA complex system is the ultimate outcome. To gain a deeper understanding of the microenvironment in the chalcone derivative complex system, synchronous spectral analysis was performed.^47^ In Fig. 7a–d, it is evident that Compound A is located near the Trp residue cutoff region. Similarly, in Fig. 7b–e, Compound B is also present in this region. However, Fig. 7c–f show no noticeable difference in the HSA–Compound C complex system compared to the other two complex systems. This indicates that Compound C may bind away from the fluorophore residues.

**Fig. 8:**
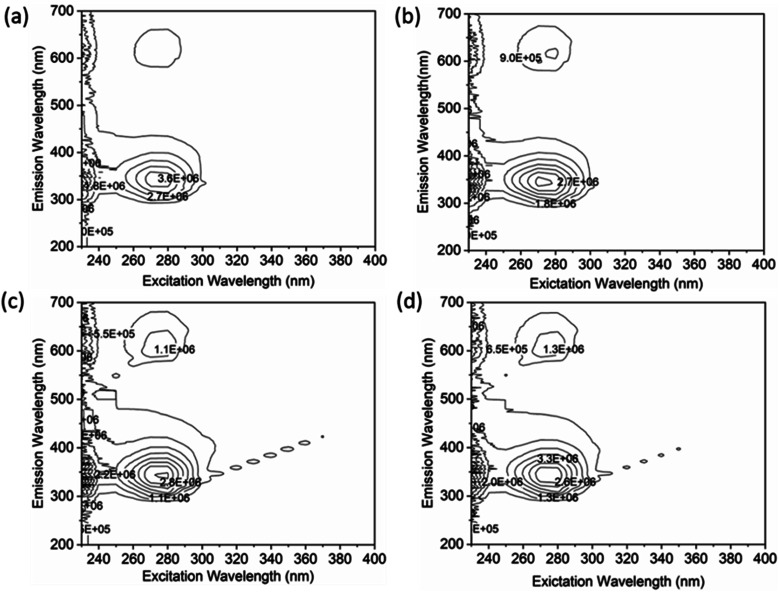
An excitation–emission matrix analysis to compare free HSA (a) with HSA complexes containing compound A (b), compound B (c), and compound C (d).

A thorough analysis of the excitation-emission matrix was conducted to understand the protein–drug complex system better and confirm the presence of drugs in the protein microenvironment.^40,41^ The study kept the concentration of HSA constant at 5 μM while chalcone derivative compounds a, b, and c were kept at a 5 μM ratio. Fig. 8a demonstrated that the maximum excitation emission intensity of free HSA molecules was around 3.6E6. On the other hand, Fig. 8b–d showed that chalcone derivative molecules had lower intensity than free HSA molecules, confirming their existence within the HSA complex system. Additionally, researchers conducted FTIR analysis to observe changes in protein molecule's secondary structure in the presence or absence of drug molecules.^50^ This analysis was based on the fact that protein molecules have more peptides and amides in the secondary structural region, making it easier to detect any biochemical changes within the protein microenvironment caused by the presence of drug molecules.

**Fig. 9:**
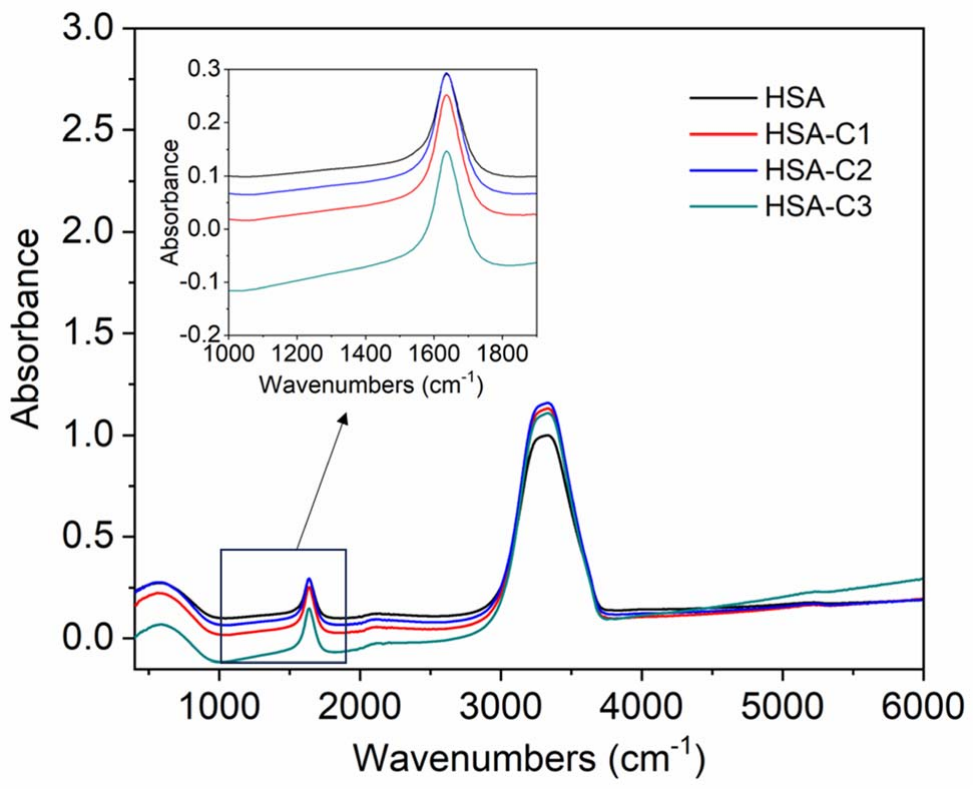
The FT-IR spectra of HSA when unbound and when bound to compound A (C1), compound B (C2), and compound C (C3). The inset shows an enlarged section of the Amide-1 region.

**Fig. 10:**
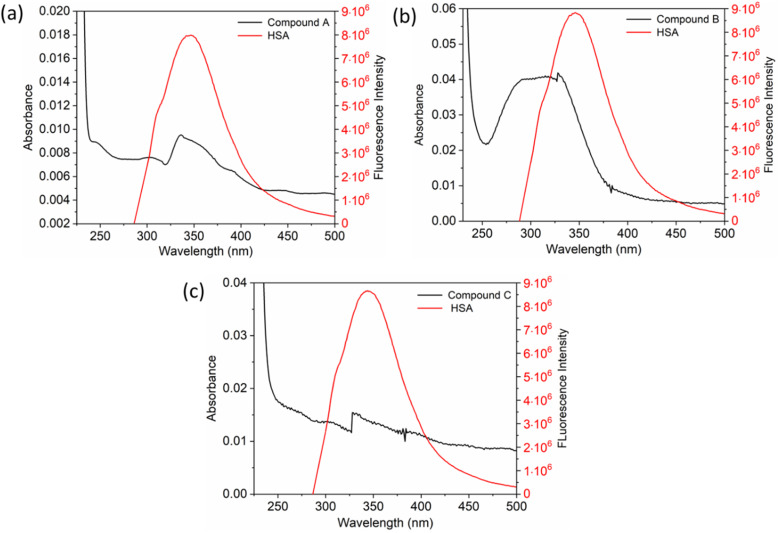
The fluorescence emission spectrum of HSA (red) overlaps with the absorption coefficient spectrum of chalcone derivatives (block) A (a), B (b), and C (c).

The inset in Fig. 9 highlights the amide I band region, where the C=O and N–H stretch can be observed. The backbone peptides have less impact in this area due to the chemical vibrations mentioned earlier. The FT-IR results indicate the presence of chalcone derivatives in the HSA complex system, with a clear decrease in the amide I band absorbance maxima upon adding these compounds. Furthermore, FRET analysis was conducted to examine the non-radiative energy distance between the acceptor and donor of the drug and protein molecule in the chalcone derivative and HSA molecule system.^49^ In the diagram labeled Fig. 10, there is an illustration of the spectrum where the emission of HSA overlaps with the absorbance of chalcone derivative molecules. From the integral overlap spectrum, calculated *J* value is 1.7283 × 10^−19^, 1.4283 × 10^−19^, 1.283 × 10^−19^ M^−1^ cm^3^, *R*_0_ is 2.128 × 10^−8^ is calculated from eqn (7), *r* = 5.5, 5.2, 4.9 nm calculated using these values *Φ*_D_ = 0.241, *k*^2^ = 2/3 *N* = 1.336, and *E* = 0.0478.

### 3.2 Computational binding analysis

**Fig. 11:**
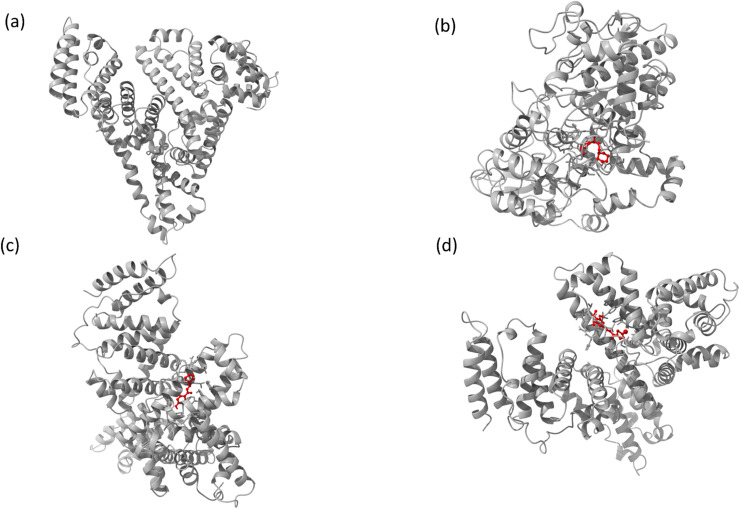
The full view of HSA (a) and the binding sites for compounds A (b), B (c), and C (d).

**Fig. 12:**
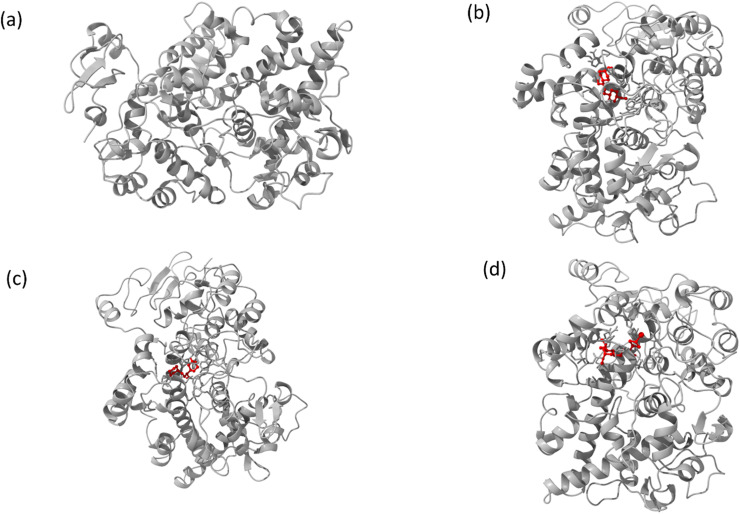
Images of (a) the full view of COX-2 and the binding site for (b) compound A, (c) compound B, and (d) compound C.

The interaction mechanism between the HSA–chalcone derivative complex system was analyzed through molecular docking, focusing on the binding process at an atomistic level.^52,54^ According to studies on structural activity relationships, chalcone derivatives are highly effective against COX-2. Therefore, we have selected carriers and target protein molecules to conduct computational binding experiments. Fig. 11 and 12 display the optimal binding position of the chalcone derivative complex in HSA and COX-2 protein molecules. The ligand plot diagram of the HSA–COX-2 complex with chalcone derivative is depicted in Fig. S1 and S2.† According to the Fig. S1† ligand plot, compound A forms three hydrogen bond connections with HSA ASP 187 (O–H⋯O), ARG 114 (O⋯N–H, O⋯N–H) residues. Compound B creates two hydrogen bond connections with SER 193 (O⋯H–O) and TYR 138 (O⋯H–O) residues.

Regarding compound C it has two hydrogen bonds that are in contact with ARG 117 (O⋯N–H, O⋯N–H) of the HSA molecule. In the COX-2 protein molecule, compound A forms a single hydrogen bond contact with PHE 504 (N–H⋯O) residue, while compound B forms three hydrogen bond contacts with MET 508 (N–H⋯O), SER 516 (N–H⋯O), and TYR 371 (N–H⋯O). Similarly, compound C forms two hydrogen bond contacts with ARG 106 (N–H⋯O) and TYR 341 (N–H⋯O) residues. Table 2 presents the values for binding energy and docking score of the protein–drug complex systems. The HSA complex system's binding energy and docking score for the chalcone derivative are as follows: compound A has a binding energy of −35.5 kcal mol^−1^ and a docking score of −5.10 kcal mol^−1^. Compound B has a binding energy of −41.82 kcal mol^−1^ and a docking score of −8.97 kcal mol^−1^. Compound C has a binding energy of −53.78 kcal mol^−1^ and a docking score of −8.17 kcal mol^−1^. The binding scores for COX-2 were observed for three different compounds, namely A, B, and C. Compound A showed a binding score of −38.63 kcal mol^−1^ and −8.81 kcal mol^−1^, while compound B displayed a score of −38.65 kcal mol^−1^ and −9.5 kcal mol^−1^. On the other hand, compound C demonstrated a high binding affinity in both protein complexes with scores of −45.6 kcal mol^−1^ and −8.71 kcal mol^−1^. Hence, further molecular dynamics simulation studies were conducted on compound C.

**Table 2:** The binding energy and docking score values for compounds A, B, and C

Protein–drug complex system	Binding energy (kcal mol^−1^)	Docking score (kcal mol^−1^)	
HSA	A	−35.5	−5.10
	B	−41.82	−8.97
	C	−53.78	−8.17
COX-2	A	−38.63	−8.81
	B	−38.65	−9.5
	C	−45.6	−8.71

**Fig. 13:**
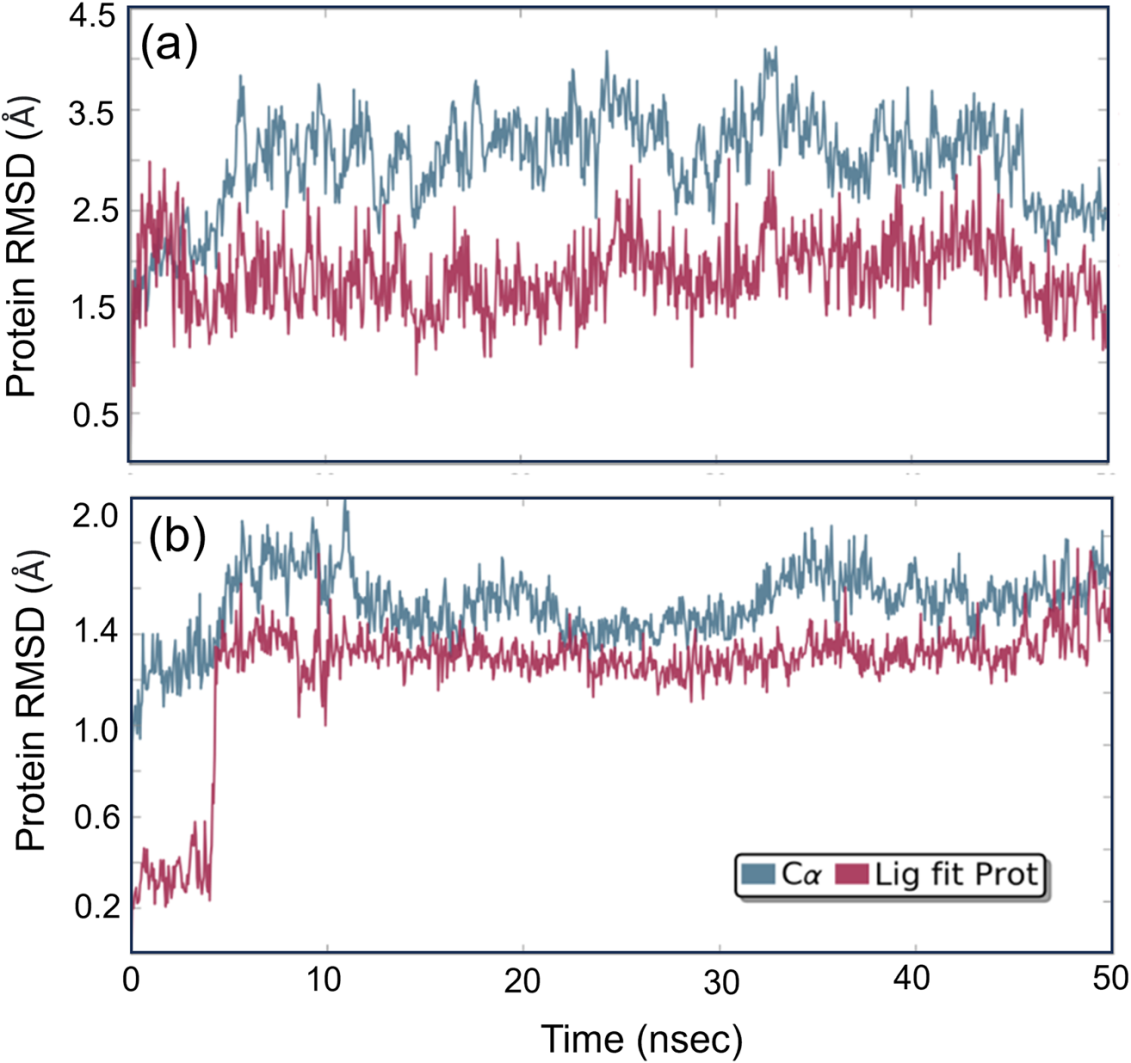
The root mean square deviation plot for compound C in both the HSA and COX-2 complex.

**Fig. 14:**
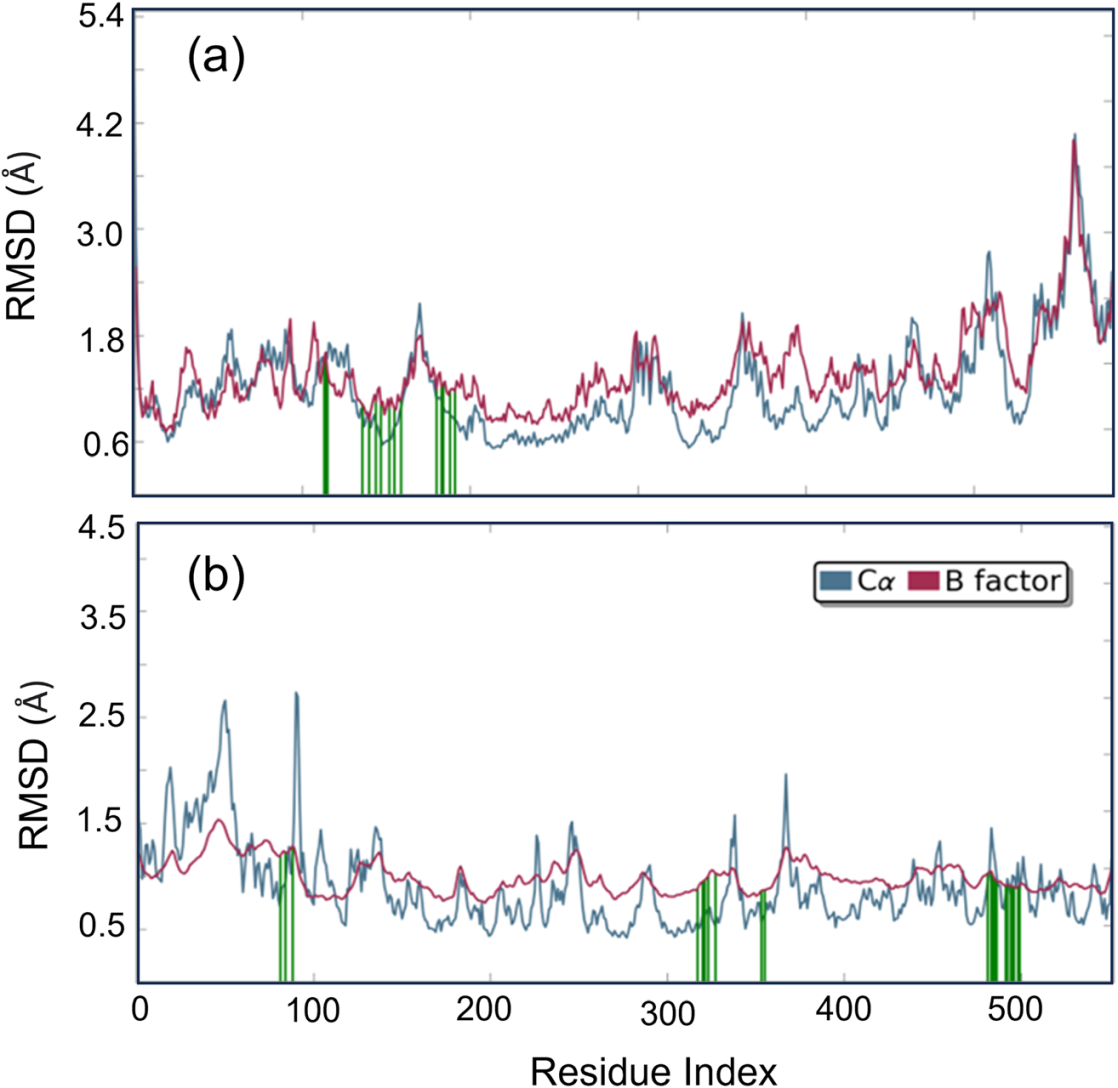
Root Mean Square Fluctuation (RMSF) plot of compound C (a) HSA (b) COX-2 complex.

Studying molecular dynamics is valuable in comprehending the stability of compounds within protein complex systems.^48–50^ To determine the convergence of the protein–drug complex system, RMSD analysis was conducted. Fig. 13 displays the RMSD plot for the free protein and the protein compound C complex. The analysis results indicate that compound C remains stable in both protein complex systems, and the systems converged within a range of 1–3 Å, suggesting that both protein–compound C complex systems undergo a more minor/globular confirmation. To gain insight into protein backbone fluctuation over a 50 ns simulation period, an RMSF plot was conducted. Fig. 14 depicts the RMSF plot of compound C within the HSA & COX-2 complex. Based on the RMSF analysis, no variation was observed in the protein–drug complexes.

Additionally, the green line depicted the active site residue interactions throughout the 50 ns simulation period. During the 50 ns simulation, we monitored the secondary structural changes, such as alpha helix and beta strands, as shown in Fig. S3.† Additionally, we plotted a timeline of the protein–drug interaction map, presented in Fig. S4.† Our results indicate that both protein–compound C maintained six contacts throughout the simulation. Based on the data presented in Fig. S5 and S6,† it is evident that the HSA–compound C complex system has 79% of H-bond contact contributed by TYR 138 residues. On the other hand, the COX-2-compound C complex system has 42% of H-bond contact contributed by PHE 504 and SER 516 residues.

In addition, we calculated the Ligand RMSF (as shown in Fig. S7†) to gain insight into how compound C interacts with the protein molecule binding environment. The findings indicate that there was no significant fluctuation of compound C in either protein–complex system throughout the 50 ns simulation period. Based on the MD results, it can be concluded that compound C remained highly stable in both the HSA and COX-2 complex systems during the simulation period.

**Fig. 15:**
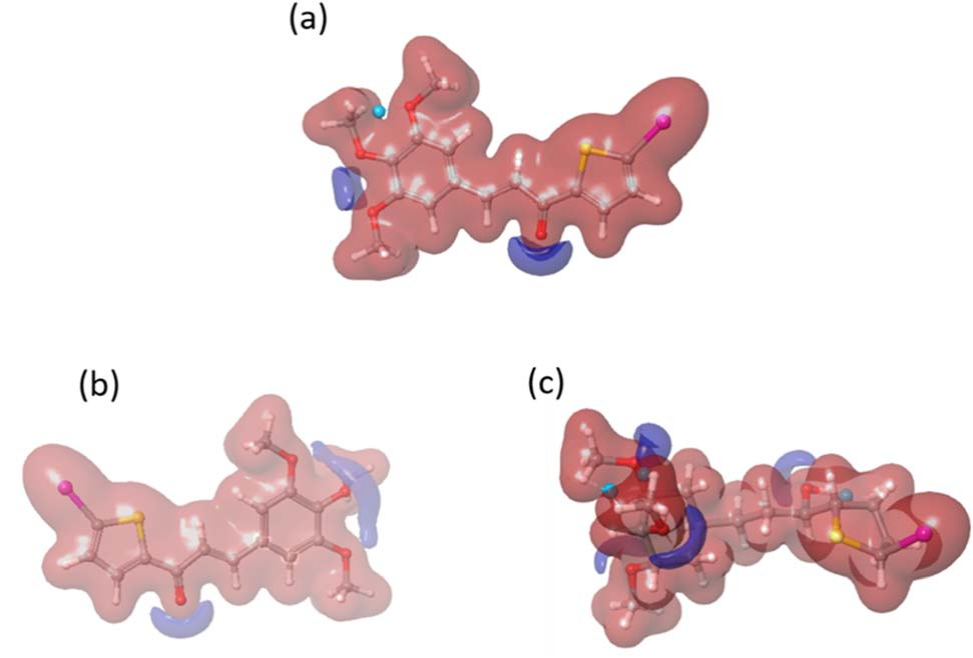
The electrostatic potential maps for compound C in different environments: (a) free compound C, (b) compound C in HSA, and (c) compound C in the COX-2 binding environment.

We conducted a DFT calculation to analyze the intermolecular interaction of compound C in its unbound state. To gather information about compound C's reactive sites in HSA and COX-2 binding environments, a single-point DFT analysis was conducted. The HOMO and LUMO values (as shown in Fig. S8† and Table 2) exhibit slight variations compared to the free compound C. According to the analysis of the electrostatic potential map in Fig. 15, there is a variation in the intermolecular interaction binding of compound C. Compared to another compound, compound C is more reactive in the COX-2 binding environment, as stated in Table S1.† Compound C effectively enhances the functionality of both protein molecules. Additionally, it exhibits superior performance in the COX-2 binding environment.^52^

## 4. Conclusion

We have synthesized chalcone derivatives and conducted binding studies with HSA using spectroscopic techniques, which are of biological interest. Additionally, the chalcone derivative exhibits a high binding affinity towards the COX-2 molecule in SAR testing. As a result, it has undergone further binding analysis using multiple computational approaches and has been compared to the HSA molecule. The presence of the chalcone derivative in the HSA complex system has been confirmed through spectroscopy analysis. The binding affinity of the chalcone derivative with both the transport protein (HSA) and target protein (COX-2) has been confirmed through rigorous molecular docking analysis. Further stability studies have been initiated for Compound C, as it has shown promising binding scores. The stability of compound C's binding in both protein molecules was confirmed through molecular dynamics investigations. Additionally, DFT analysis revealed the reactive site of compound C in both proteins. Interestingly, it was found that compound C was more reactive in the COX-2 binding environment when compared to HSA.

## Conflicts of interest

There are no conflicts to declare.

## Supplementary Material

RA-014-D3RA07438B-s001
