# Supplementary material for: Chalcone derivatives' interaction with human serum albumin and cyclooxygenase-2
Source: RSC Adv. 2024 Jan 17;14(4):2835–49. doi: 10.1039/d3ra07438b (PMC10792617; doi:10.1039/d3ra07438b)
Supplement: RA-014-D3RA07438B-s001 [file RA-014-D3RA07438B-s001.pdf]

Electronic Supplementary Material (ESI) for RSC Advances.
This journal is © The Royal Society of Chemistry 2024

# Supporting Information

# Chalcone derivatives binding with human serum albumin and cyclooxygenase-2

Subramani Karthikeyan[a*], Shanmugavel Chinnathambi[b*], Ayyavu Thirunarayanan[c], Leon Bernet Shano[d], Arasappan Hemamalini[e], Anandh Sundaramoorthy[f], Rajendiran Mangaiyarkarasi[g], Norhidayah Abu[h], Singaravelu Ganesan[f], Ganesh N. Pandian[h*]

[a]Centre for Healthcare Advancement, Innovation and Research, Vellore Institute of Technology, Chennai, 600 127, India

[b]Institute for Integrated Cell-Material Sciences, Institute for Advanced Study, Kyoto University, Kyoto, 616-8510 Japan

[c]Department of Chemical Engineering Biotechnology and Materials, FCFM, University of Chile, Av. Beauchef 851, Santiago, Chile

[d]Division of Physics, School of Advanced Sciences, Vellore Insititute of Technology (VIT) Chennai campus, Vandalur-Kelambakkam Road, Tamil Nadu 600127, India

[e]Department of Chemistry, Government College of Engineering Srirangam, Sethurapatti, Thiruchirappalli, Tamil Nadu, India

[f]Department of Medical Physics, Anna University, Chennai-600 025, India.

[g]Centre for Nanotechnology Research, Vellore Institute of Technology, Vellore, Tamil Nadu, India

[h]Department of Medical Microbiology & Parasitology, School of Medical Sciences, Universiti Sains Malaysia, Health Campus, Kubang Kerian 16150, Kelantan, Malaysia

Corresponding email address:

s.karthikeyan@vit.ac.in

chinnathambi.shanmugavel.8s@kyoto-u.ac.jp

namasivayam.ganeshpandian.5z@kyoto-u.ac.jp

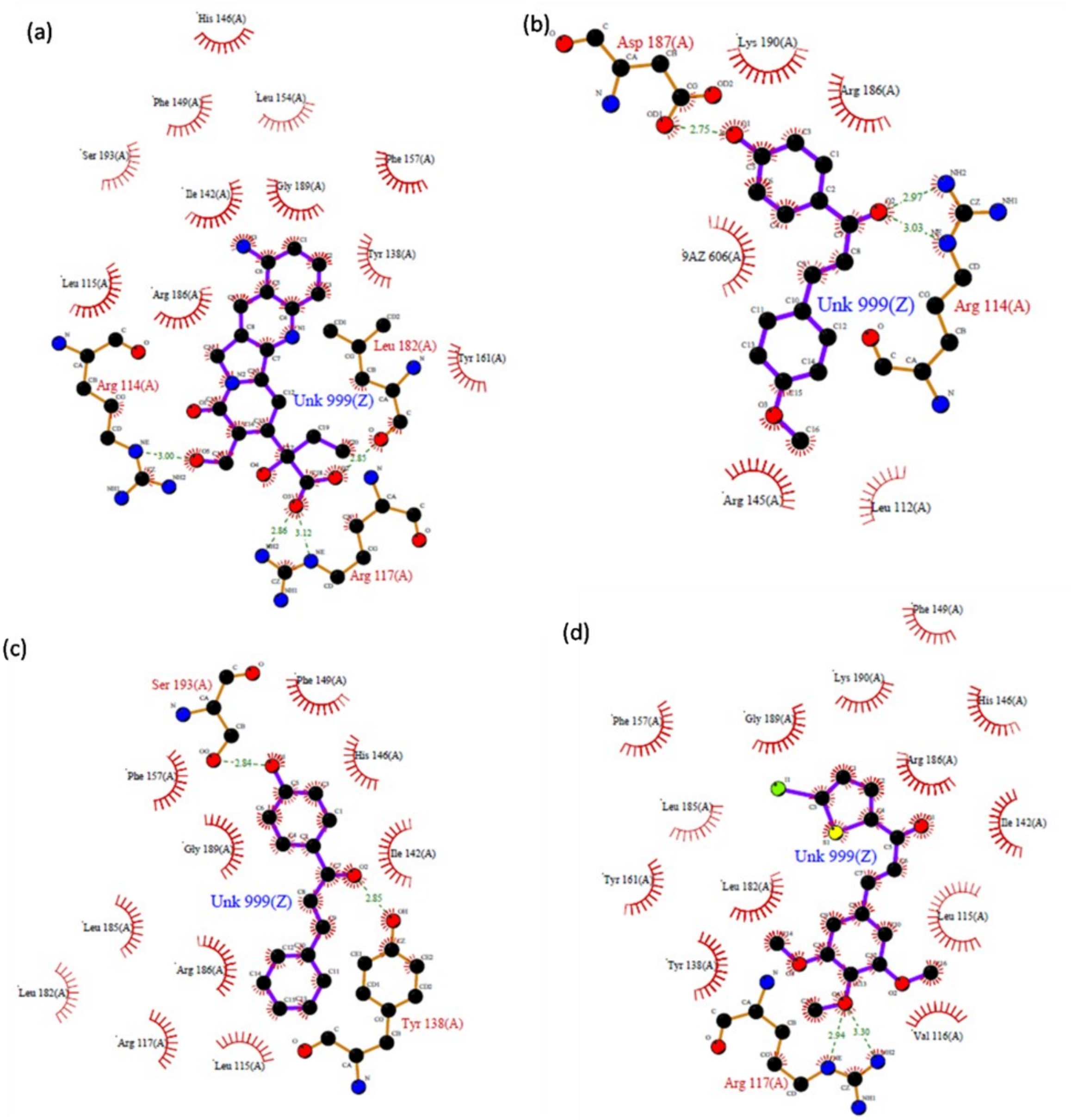


**Figure.S1** shows the 2D ligand plot in HSA binding site (a) co-crystal site (b) compound A (c) compound B (d) compound C.

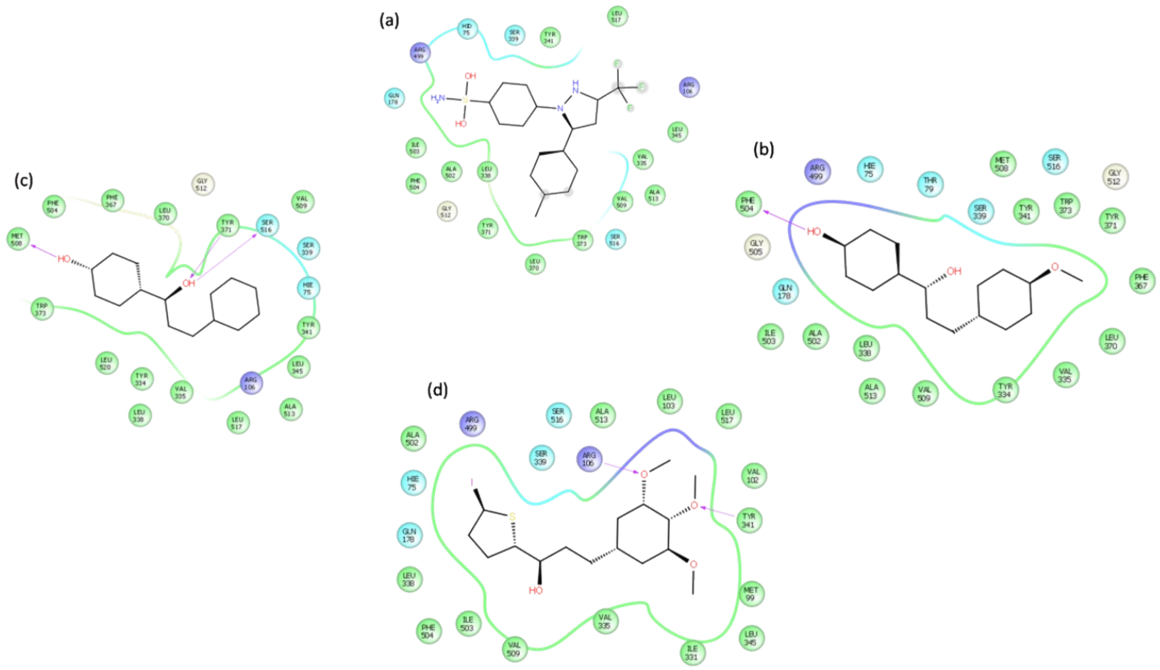


**Figure.S2** shows the 2D ligand plot in COX-2 binding site (a) co-crystal site (b) compound A (c) compound B (d) compound C.

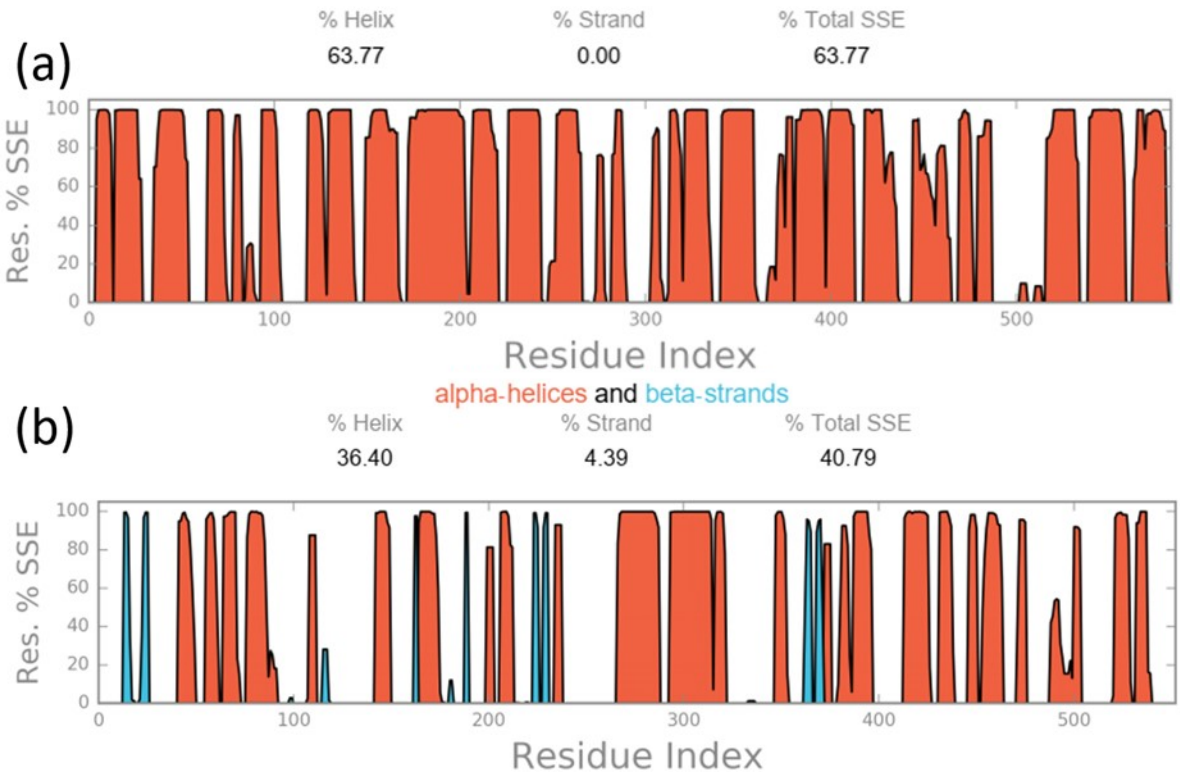


**Figure.S3** Protein secondary structure elements analysis plot of compound C in (a) HSA (b) COX-2 complex system.

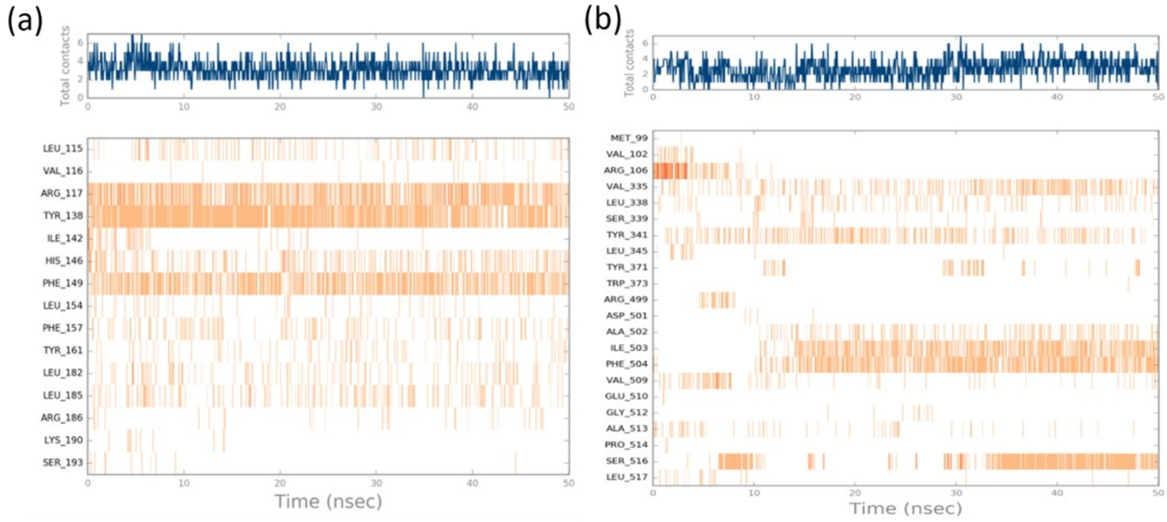


**Figure.S4** Total contacts of compound C in (a) HSA (b) COX-2 complex system.

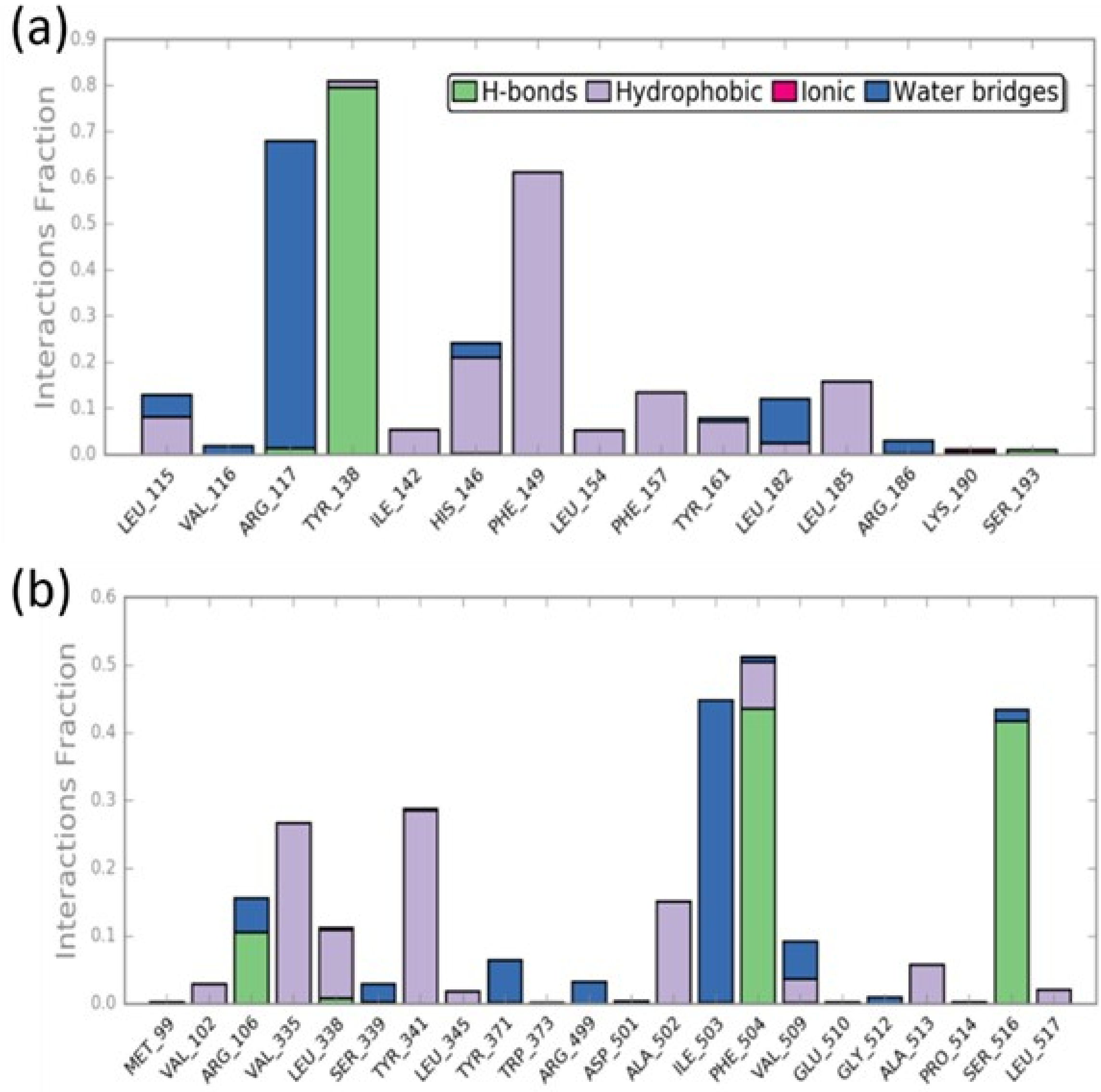


**Figure.S5** Protein-ligand interaction fraction plot of compound C in (a) HSA (b) COX-2 complex system.

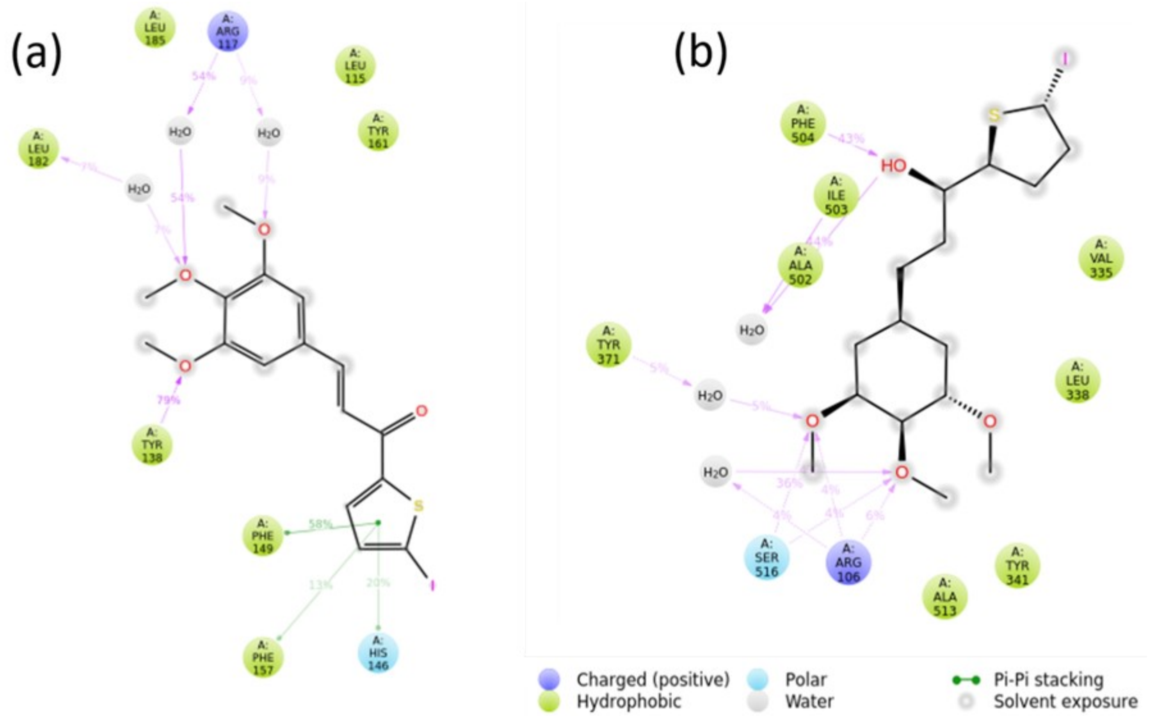


**Figure.S6** shows MD ligand plot of compound C in (a) HSA (b) COX-2 complex system.

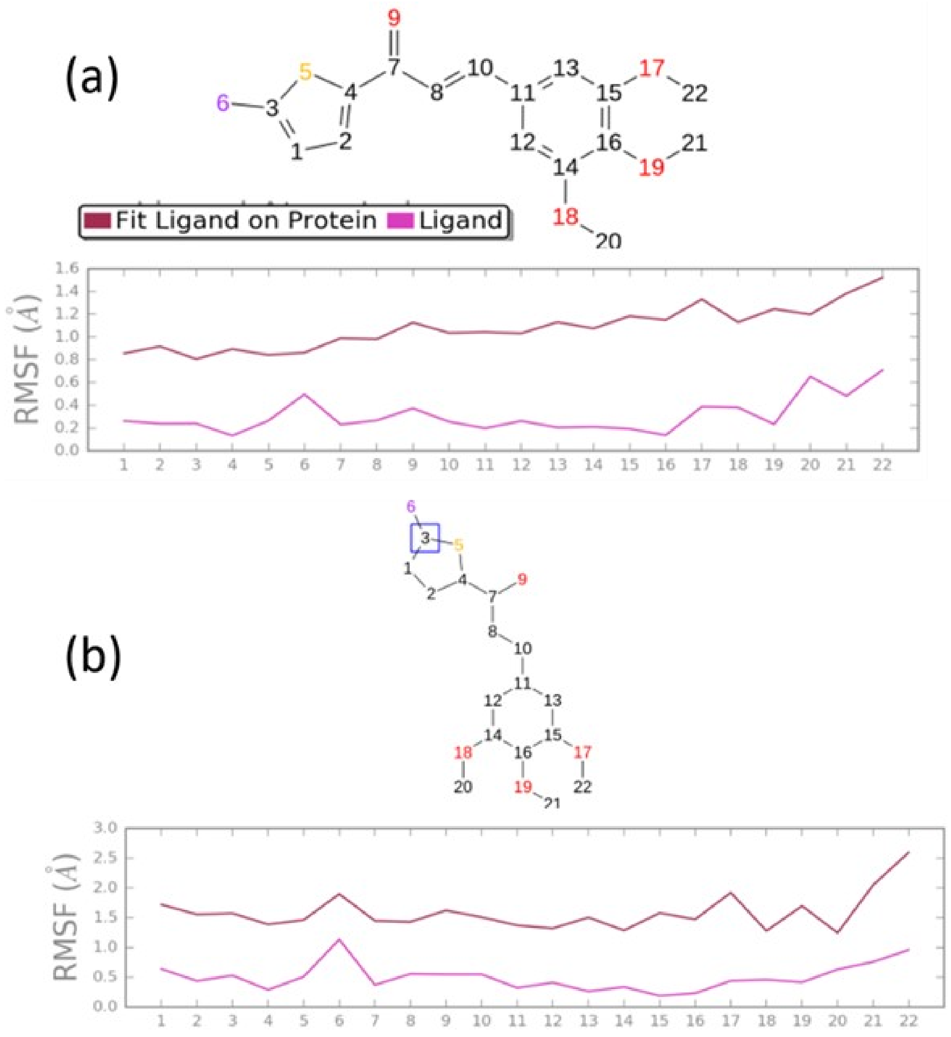


**Figure.S7** shows Ligand RMSF plot of compound C in (a) HSA (b) COX-2 complex system.

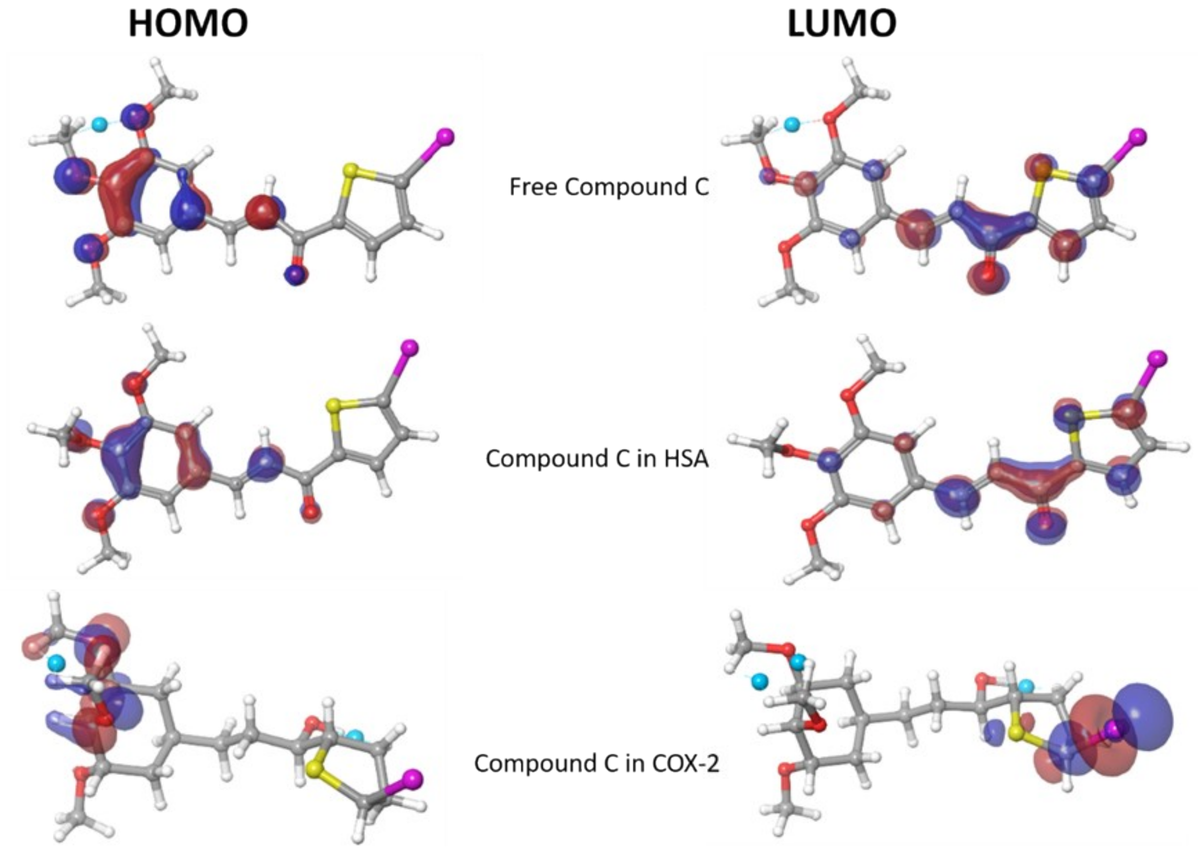


**Figure.S8** HOMO-LUMO plot of free compound C and compound C in HSA and COX-2 binding environment.

**Table S1.** HOMO-LUMO parameters of free compound C and compound C in HSA and COX-2 binding environment.

| DFT Globalised Parameter | Compound C | Compound C in HSA | Compound C in COX-2 |
|---|---|---|---|
| HOMO (Hatree) | -0.192037 | -0.211642 | -0.214717 |
| LUMO (Hatree) | -0.073375 | -0.078166 | -0.037280 |
| ALIE (kcal/mol) | 200.77 | 196.918 | 182.579 |
| ESP | -32.63 | -32.29 | -33.42 |
| Interaction Strength ($e^{-}/a_0$**$^3$) | -0.05 | -0.05 | -0.05 |
